# Genome Sequencing: Using Models to Predict Who's Next

**DOI:** 10.1371/journal.pbio.0030025

**Published:** 2005-01-04

It's hard to believe it was just ten years ago that scientists reported the first complete genome sequence of an organism, the bacterial pathogen Haemophilus influenzae. The list has grown considerably since then: add over 160 bacterial species (and counting), most major model organisms, and an ever-growing list of mammals—including, of course, humans. With 99% of our genome now fully sequenced, the Human Genome Project's next major goal is to identify all the functional elements contained in our 2.85 billion nucleotides. Such an effort is hardly trivial: producing the sequence of a mammalian-size genome can run from $10 to $50 million, the estimated price tag of the Cow Genome Project.

In an ideal world, any organism would be fair game for sequencing, but in the real world, sequencing resources are scarce. Comparing genome sequences turns out to be a great way to identify regions that have important functions, but comparative genomics studies would be far more efficient if scientists could figure out in advance which genomes would reveal the most information about a particular question. Taking up that challenge, computational biologist Sean Eddy reports a statistical model that predicts how many genomes, and at what evolutionary distance, are needed for effective comparative genomic analyses. In addition to confirming some working principles of comparative genomics, the model also reveals a surprisingly simple guideline for future studies.

Comparative genomics works by aligning sequences of different organisms to identify patterns that operate over both large and small distances. Aligning mouse chromosomes with human chromosomes, for example, shows that 99% of our protein-coding genes align with homologous sequences in mice. Underlying such analyses is the principle that DNA sequences that are highly conserved are likely to be functionally important. A common assumption is that adding more comparative genomes to the alignment helps distinguish functionally significant from irrelevant conserved sequences.

How do you go about creating an abstract model that captures what Eddy calls the “essential flavor of comparative genomic analysis”? His model puts aside the specific characteristics of individual organisms, genomic features, and analysis programs in favor of identifying higher-level patterns and scaling relationships, specifically between the number of genomes, evolutionary distance, and feature size (features include genetic elements like exons and transcription factors).

The model shows that the number of genomes required to identify conserved regions—that is, regions evolving under selection—scales inversely with the size of the feature being sought. Thus, to look for conserved sequences half as long, you need twice as many genomes, assuming a constant evolutionary distance and statistical power. For example, to identify a conserved human feature the size of a coding exon (about 50 nucleotides), it is sufficient to compare just the human and mouse genomes. But to identify conserved single nucleotides, you would need 55 comparative genomes at “mouse-like” evolutionary distances (roughly 75 million years).

Things get a little trickier when varying evolutionary distance. We can see a substitution only at a given point in time: we can't tell how many times a site has changed, for example, or whether it changed at some point and then changed back. But at short evolutionary distances—where it's safer to assume no sites have changed more than once—the evolutionary distance is roughly the same as the fraction of sites identified as changed, and evolutionary distance and the number of genomes needed scale inversely. Therefore, the closer the evolutionary distance, the more genomes needed: one would need seven times as many comparative genomes using human/baboon distances, for example, compared to human/mouse distances. So when it comes to using primate sequences to study the human genome, our most distant relatives (such as lemurs) offer far more comparative analysis power than our next of kin (chimps and bonobos).

While this model confirms the intuitive assumption that identifying smaller features requires more genomes, it reveals an inverse scaling relationship far more direct, and precise, than previously imagined. With the next phase of the Human Genome Project under way, Eddy's model offers valuable guidelines for identifying which genomes and how many might best meet this ambitious goal.

